# Full-Genome Sequences of Influenza H3N2 Virus Strains Isolated from Finnish Patients during the 2012-2013 Epidemic Season

**DOI:** 10.1128/genomeA.00039-14

**Published:** 2014-03-27

**Authors:** Triin Lakspere, Hannimari Kallio-Kokko, Anu Kantele, Pirkko Mattila, Henrikki Almusa, Denis Kainov, Laura Kakkola

**Affiliations:** a The Institute for Molecular Medicine Finland (FIMM), Helsinki, Finland; b Helsinki University Hospital Laboratory, HUSLAB, Helsinki, Finland; c Helsinki University Central Hospital, HUCS, Helsinki, Finland

## Abstract

Here, we sequenced 10 influenza A(H3N2) virus genomes isolated from Finnish patients diagnosed with flu-like illness during the 2012-2013 influenza season. The alignment showed a high number of amino acid substitutions (238 in total) in only 10 samples, proving that a high mutation rate exists in seasonal influenza A viruses.

## GENOME ANNOUNCEMENT

The *Orthomyxoviridae* are a family of RNA viruses, with the most substantial member being influenza A virus (IAV) (1). The IAV genome consists of 8 single-stranded RNA (ssRNA) segments that encode 12 proteins: hemagglutinin (HA), neuraminidase (NA), M proteins (M1 and M2), nucleocapsid protein (NP), nonstructural proteins (NS1 and NS2), and polymerase subunits (polymerase acidic [PA], PA-X, polymerase basic 1 [PB1], PB1-F2, and PB2). IAVs are subtyped based on surface glycoprotein sequences of HA (H1 to H17) and NA (N1 to N9) (1, 2).

We report here the whole-genome sequences of 10 influenza A(H3N2) viruses isolated from nasopharyngeal aspirate (NPA) samples from Finnish patients during the 2012-2013 influenza season. The sequences were obtained (100-bp reads, average 2,791,276 reads/isolate) and analyzed as described previously (3, 4). The analysis of the genomes revealed in total 1,024 nucleotide and 238 amino acid substitutions compared to the reference A/Sydney/5/1997 strain. The viral surface proteins HA and NA were highly mutated (59 and 49 amino acid substitutions, respectively). All 10 isolates had an amantadine resistance mutation (S31N) in the viral M2 protein, and none had an H3N2 subtype-specific oseltamivir resistance mutation (E119V) in the viral NA protein (5). Interestingly, 50% of the seasonal strain sequences had a mutation in the PB2 protein (E627K) that has been related to high virulence in avian influenza viruses (6). The full-length sequences of these H3N2 isolates could be used in understanding the evolution of H3N2 viruses and also aid in vaccine development.

### Nucleotide sequence accession numbers.

Ten whole-genome sequences of H3N2 isolates from Finland from the 2012-2013 season have been deposited in Genbank under accession no. KF805640 to KF805705.
